# A New Long-Term Downward Surface Solar Radiation Dataset over China from 1958 to 2015

**DOI:** 10.3390/s20216167

**Published:** 2020-10-29

**Authors:** Ning Hou, Xiaotong Zhang, Weiyu Zhang, Jiawen Xu, Chunjie Feng, Shuyue Yang, Kun Jia, Yunjun Yao, Jie Cheng, Bo Jiang

**Affiliations:** 1 State Key Laboratory of Remote Sensing Science, Faculty of Geographical Science, Beijing Normal University, Beijing 100875, China; houning_0110@mail.bnu.edu.cn (N.H.); zhangweiyu@mail.bnu.edu.cn (W.Z.); xujw@mail.bnu.edu.cn (J.X.); fcj20190901@mail.bnu.edu.cn (C.F.); ysy_0128@mail.bnu.edu.cn (S.Y.); jiakun@bnu.edu.cn (K.J.); yaoyunjun@bnu.edu.cn (Y.Y.); Jie_Cheng@bnu.edu.cn (J.C.); bojiang@bnu.edu.cn (B.J.); 2 Beijing Engineering Research Center for Global Land Remote Sensing Products, Institute of Remote Sensing, Science and Engineering, Faculty of Geographical Science, Beijing Normal University, Beijing 100875, China

**Keywords:** downward surface shortwave radiation, extremely randomized trees, random forest, global brightening, global dimming

## Abstract

Downward surface solar radiation (*Rs*) plays a dominant role in determining the climate and environment on the Earth. However, the densely distributed ground observations of *Rs* are usually insufficient to meet the increasing demand of the climate diagnosis and analysis well, so it is essential to build a long-term accurate *Rs* dataset. The extremely randomized trees (ERT) algorithm was used to generate *Rs* using routine meteorological observations (2000–2015) from the Climate Data Center of the Chinese Meteorological Administration (CDC/CMA). The estimated *Rs* values were validated against ground measurements at the national scale with an overall correlation coefficient value of 0.97, a mean bias of 0.04 Wm^−2^, a root-mean-square-error value of 23.12 Wm^−2^, and a mean relative error of 9.81%. It indicates that the estimated *Rs* from the ERT-based model is reasonably accurate. Moreover, the ERT-based model was used to generate a new daily *Rs* dataset at 756 CDC/CMA stations from 1958 to 2015. The long-term variation trends of *Rs* at 454 stations covering 46 consecutive years (1970–2015) were also analyzed. The *Rs* in China showed a significant decline trend (−1.1 Wm^−2^ per decade) during 1970–2015. A decreasing trend (−2.8 Wm^−2^ per decade) in *Rs* during 1970–1992 was observed, followed by a recovery trend (0.23 Wm^−2^ per decade) during 1992–2015. The recovery trends at individual stations were found at 233 out of 454 stations during 1970–2015, which were mainly located in southern and northern China. The new *Rs* dataset would substantially provide basic data for the related studies in agriculture, ecology, and meteorology.

## 1. Introduction

The downward surface solar radiation (*Rs*) plays a dominant role in the global radiation budget as it is a basic element of energy source on the earth [1,2]. It is an important driving force of various biological, chemical, and physical processes of the Earth’s system [3,4,5,6]. Therefore, understanding and determining the variability of *Rs* are crucial for practical applications such as environmental, hydrological, and ecological studies [7,8,9,10,11].

*Rs* is not directly measurable using the satellite sensors due to the atmospheric influences, the introduction of empirical or physical-based models for *Rs* estimation will induce possible uncertainties [12]. Direct ground measurements are essential for *Rs* quantification since it is one of the most accurate *Rs* data sources. The monitoring of *Rs* started at limited ground stations since the early 20th century, the measurements of *Rs* were more widespread after the International Geophysical Year (1957/1958) [13]. The worldwide ground measurements of *Rs* revealed a significant declining trend from the 1950s to the 1980s (coined as ‘global dimming’), as well as a recovery since the late 1980s phenomenon (‘global brightening’) in different regions around the world [14,15,16,17,18], such as Europe [19,20], USA [21,22], New Zealand [23], and Japan [24]. For example, it reported that a decline of *Rs* (−2.0 to −3.1 Wm^−2^ per decade) during 1971–1986 and a recovery of *Rs* (1.1 to 1.4 Wm^−2^ per decade) during 1987–2002 were found in Europe [19].

Many studies have also widely reported that the *Rs* in China experienced a significant decline from the 1960s and a subsequent recovery from the 1990s [25,26,27,28,29,30,31,32]. Che et al. [29] found the *Rs* over China exhibited a significant decline (−4.5 Wm^−2^ per decade) during 1961-2000 using the data at 64 stations from the Climate Data Center of the Chinese Meteorological Administration (CDC/CMA). A decreasing tendency in *Rs* (−3.1 Wm^−2^ per decade) was found based on the analysis at 85 CDC/CMA stations by Qian et al. [30]. Wang et al. [32] noticed that the *Rs* in China declined (8.1 Wm^−2^ per decade) during 1961–1989 and increased (2.7 Wm^−2^ per decade) during 1990–2009 using the ground measured *Rs* data at 52 CDC/CMA stations. Yang et al. [25] reported the *Rs* in China exhibited a decline (−4.9 Wm^−2^ per decade) during 1958-2016 based on the *Rs* measurements from 95 CDC/CMA stations. Although much effort has been conducted on the long-term *Rs* variability analysis over China, it still has fierce controversy. Moreover, the number of stations used in these studies was limited. Thus, it is still essential to reconstruct the long-term accurate *Rs* dataset at more stations for analyzing the variations of *Rs* over China.

It is known that *Rs* is influenced by temporal inhomogeneities due to the updates in the measuring in instrumentation and relocation [33,34]. The changes in the measuring equipment of the CDC/CMA may result in uncertainty of the long-term trend analysis of the *Rs* over China by introducing breaks [35,36,37]. The *Rs* is closely related to meteorological variables, including clouds, aerosols, temperature, precipitation, sunshine duration, and other factors [38,39,40,41,42,43,44]. It has been proved that it is feasible to reconstruct *Rs* dataset from those widely measured meteorological variables. Compared to the radiation observation data, the meteorological measurements have better temporal homogeneity and spatial representativeness. Currently, various methods have been widely applied to estimate *Rs* using meteorological variables, which include the empirical statistical models [45,46], physical parameterization models [42,47], and machine learning methods [38,40,48,49]. The empirical statistical models establish relationships between the meteorological variables and *Rs* observations for estimating *Rs*, but they are usually site-dependent. The physical parameterization models simulate the interactions between the solar radiation and atmosphere for estimating *Rs*. However, the proposed physical parameterization models may be directly influenced by the accuracy of the input variables, such as clouds and aerosol properties, and so on [50,51,52].

The machine learning method (e.g., artificial neural network (ANN), support vector machine (SVM), Random Forest (RF), etc.), which automatically learns rules from a large amount of historical data for prediction, is an alternative approach to reconstruct *Rs* accurately. For example, Tang et al. [40] applied an ANN model to develop a 50-year dataset of daily *Rs* over China, the new *Rs* data showed higher accuracy than previous estimated *Rs* data. Fan et al. [53] applied the SVM and extreme gradient boosting (XGBoost) to estimate daily *Rs* over China, it showed the XGBoost method was promising and effective to estimate *Rs*. Wang et al. [54] evaluated the Adaptive Neuro Fuzzy Inference System (ANFIS) and M5 model tree (M5Tree) method for estimating daily *Rs* in China, it indicated the ANFIS method had better performance than the M5Tree method in *Rs* estimation. Some studies also applied the machine learning methods for estimating *Rs* using satellite observations [55,56,57,58,59,60]. As far as now, RF has been widely used in remote sensing for classification and regression problems [55,56,61,62] due to its great accuracy and efficiency. Extremely randomized trees (or extra trees, ERT), considered as the further development of the RF method, can efficiently reduce the variance of models and excavate more significant information than the RF method by introducing a stronger randomization method [63]. However, studies on the *Rs* estimation based on the ERT method is rare compared to that based on the RF method.

Therefore, the main objective of this study was to develop an *Rs* estimation model based on the ERT method using quality-controlled radiation measurements and meteorological measurements from CDC/CMA. The *Rs* estimates based on the ERT approach were compared with ground measurements. Additionally, the spatial distributions, seasonal variations, and long-term trends of the estimated *Rs* over China from 1970 to 2015 were also analyzed based on the new dataset. Section 2 and Section 3 introduce the data and methods used in this study. Section 4 introduces the results of the ERT-based model and presents the spatiotemporal analysis of the *Rs* estimates over China. Section 5 discusses the probable cause of variations in *Rs*. A short summary is given at the end of this paper.

## 2. Data

There is a total of 756 meteorological stations in China and only 96 meteorological stations have records of solar radiation since 1994. The *Rs* measurements by CDC/CMA started in 1957, and it is noted that the radiometers equipped at CDC/CMA stations had been updated during 1990–1993. Before releasing the radiation data, a quality control process was conducted by CDC/CMA. Nevertheless, some studies [43,64] suggested that the radiation data of CDC/CMA need to be inspected more strictly for further application. The method proposed by Tang et al. [43] was performed to examine the quality of radiation data. In this study, the “complete records” of meteorological data, which was defined as data that contains more than 20 days in every month, and 12 months in a year, were used for model construction at 96 CDC/CMA stations and reconstructing *Rs* at 756 CDC/CMA stations. To analyze the temporal variations of *Rs* in China, the reconstructed *Rs* data at 454 CDC/CMA stations with complete records for consecutive 46 years (1970–2015) were used for analysis.

The routine daily meteorological measurements, which include air pressure, air temperature, wind speed, relative humidity, daily precipitation, water vapor pressure, sunshine duration, and the *Rs* data collected from CDC/CMA were used to reconstruct *Rs*. In addition, the temporal information was also used to reduce the influence of the seasonal cycle of *Rs,* since *Rs* have the clear temporal variations. For each station, the cosine of the radian difference (*T*) is obtained according to Equation (1) proposed by Wei et al. [65], which is capable of minimizing the influence of the seasonal cycle.

(1) $$T_{t}=\cos\left(2\pi \frac{d_{t}}{D}\right)$$

where *d* represents the Julian day of the year (DOY), *D* denotes the total number of days in a year.

**Figure 1:**
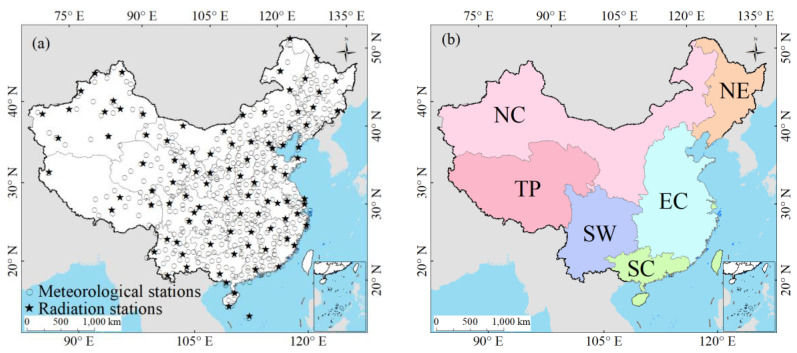
(**a**) The spatial distribution of 454 Climate Data Centers of the Chinese Meteorological Administration (CDC/CMA) stations, the 96 radiation stations are denoted by five-pointed star symbols, (**b**) six climatic regions of China.

Figure 1a displays the spatial distributions of 454 stations from CDC/CMA, the 96 radiation stations were denoted by the star symbols. This study also discussed the regional *Rs* trends in different climatic regions of China. Various approaches for classifying climatic types in China [66,67,68,69] were presented in previous studies. In this study, six climatic regions in China were classified according to the classification in Wang et al. [68] and Zhou et al. [69]. Figure 1b displays the six climatic zones, northeast China (NE), north China (NC), east China (EC), south China (SC), southwest China (SW), and Tibetan Plateau (TP).

## 3. Methodology

### 3.1. Extremely Randomized Trees (ERT)

The decision tree, a nonlinear and nonparametric method, is widely used in regression and classification problems. Decision trees predict the value of a target variable using a set of values of input variables. The decision trees are capable of dealing with problems of large-scale data using plenty of training samples and input variables. The decision tree method is easy and clear to understand. The relevant variables can be recognized during the growth of trees, which provides robustness for the decision trees model [63]. However, the high sensitivity for training samples is the main inadequacy of the single tree [70,71], the low accuracy and high randomness of the single tree result from this high sensitivity restrict the application of the single tree, particularly in handle numerous datasets [63]. The ensemble of trees, such as RF and ERT, is capable of conquering the problem of single tree models.

RF, a powerful ensemble-learning method, was proposed by Breiman in 2001, which is widely applied as a classification and regression tool [72]. The RF method employs the bootstrap technique (Ibrahim and Khatib 2017), the bootstrap samples are randomly created and replaced from the training data. Using bootstrap samples in the RF model generates around one-third unused subset, named as out-of-bag data (OBB) [72], the rest data are called in-bag data. According to the minimized Gini index, the best split is determined among the subgroup of the random selection at each node.

ERT, a tree-based machine learning method, was proposed by Geurts et al. [73]. ERT is considered as the further development of the RF method. ERT has been widely used to solve diverse sequence-based prediction problems [74,75]. ERT introduces a more powerful randomization method to efficiently reduce the variance of models and excavate more significant information than other tree-based methods. There are three main parameters in ERT for regression problems including K, n_min,_ N, and M. The K denotes the number of random splits, the n_min_ indicates the minimum sample to split a node [63,73], the N is size of samples, and the M represents the number of trees of ERT. The growth of trees in ERT is conducted through exactly defining values of the K on each node to achieve pure outputs in all subsamples [63], the M controls the degree of variance reduction in the ensemble model [73]. In addition, the variance of ERT generally further decreases with the increase of M [63], but the bias may increase slightly. The ERT offers added robustness about obvious errors due to the marginal affection of outliers on ERT prediction. The variable importance measure is also provided by the ERT method, which is defined as follows:

(2) $$VI^{\left(t\right)}\left(z\right)=\frac{1}{N}\sum_{N}\left(\frac{\sum_{X_{a}\in B^{c\left(t\right)}}\left(I\left(L_{b}=c_{a}^{\left(t\right)}\right)-I\left(L_{b}=c_{a,\pi z}^{\left(t\right)}\right)\right)}{\left|B^{c\left(t\right)}\right|}\right)$$

where $B^{c\left(t\right)}$ is the OBB sample for a tree, $X_{a}$ is sample value, *t* is the tree number, $c_{a}^{\left(t\right)}$ and $c_{a,\pi z}^{\left(t\right)}$ are the predicted values before and after change of variables, *a* and *b* are the number of samples per leave and per tree, respectively.

The ERT and RF methods are similar but different in two aspects. The ERT employs all training samples instead of the bootstrap algorithm during the growth procedure; and the ERT performs node splitting by random selections of cut-points, instead of the best node split based on the Gini index in the RF [76], which is calculated according to Equation (3):

(3) $$I_{n}=1-{\sum_{i=1}^{K}\left(\frac{M_{i}}{N}\right)}^{2}$$

The ERT method generate a set of independent decision trees based on the features space F. In this study, the features space X = {air pressure, air temperature, wind speed, relative humidity, water vapor pressure, daily precipitation, sunshine duration, elevation, and cosine of the radian difference}. The followed Algorithm 1 illustrates the procedure. At the begining of the training stage, the ensemble tree set is initialized as empty. Then, each decision tree is built with randomly selected features without replacement. During the testing stage, a predicted value $y_{i}$ is obtained by each decision tree. The final result is the average of all the decision trees.
**Algorithm 1** Extremely Randomized Trees Algorithm [73]Given ($x_{1},y_{1}$), ($x_{2},y_{2}$), …, ($x_{n},y_{n}$), with features space F where ($x_{i},y_{i}$) ∈ (X,Y) training data. Given the N and max_depth (max depth of each tree) **procedure** Train (($x_{1},y_{1}$), ($x_{2},y_{2}$), …, ($x_{n},y_{n}$))   ERT← {}   **for**
*i* from 1 to *N* do     decision tree $T_{i}\in X\to Y$     **while** (do depth (T)) < max_depth)      Random select $X_{i}\subset X$ without replacement      Random select feature f∈F       Use f as node to construct tree     **end while**     $ERT=ERT\cup \left\{T_{i}\right\}$
    **end for**     **return**
ERT **end procedure** **procedure** Test (*x*) for *i* from 1 to *N* do  Select $T_{i}$ from ERT   $y_{i}\leftarrow T_{i}\left(x\right)$  **end for**  $y=\frac{\sum_{i=1}^{N}y_{i}}{N}$ **return** y**end procedure**

### 3.2. Mann–Kendall (M-K) Test

The nonparametric Mann–Kendall (M-K) test [77,78] was employed for detecting the significant temporal variation of *Rs*. It is a rank-based method to identify tendencies of time-series dataset. The relative magnitudes of the samples are compared by the M-K test rather than the data values themselves. In addition, the M-K test does not require that samples conform to a certain type of distribution. The M-K test is an effective approach to identify tendencies of meteorological and other relevant variables without being affected by certain data distribution and outliers. The M-K statistics *s* is defined as:

(4) $$s=\sum_{m=2}^{i}\sum_{n=1}^{m-1}f(T_{m}-T_{n})$$

where i is the number of data. $T_{m}$ and $T_{n}$ (m>n) are observations in time series. f() is given as:

(5) $$f\left(T_{m}-T_{n}\right)=\left\{\begin{array}{cc} 1 & T_{m}-T_{n}>0\\ 0 & T_{m}-T_{n}=0\\ -1 & T_{m}-T_{n}<0 \end{array}\right\}$$

The variance of statistics *s* is given as:

(6) V(s)=i(i−1)(2i+5)/18

The standardized test statistics *Z* is given as:

(7) $$Z=\left\{\begin{array}{cc} (s-1)/\sqrt{V(s)} & s>0\\ 0 & s=0\\ (s+1)/\sqrt{V(s)} & s<0 \end{array}\right\}$$

A positive Z value represents the increasing trend and a negative value represents the decreasing trend. The significance levels (p=0.05 and 0.01) are used to identify the significant trend, corresponding $Z_{1-\alpha /2}=\pm 2.58$ and $Z_{1-\alpha /2}=\pm 1.96$, respectively.

## 4. Results and Analysis

Previous studies [35,36,37] reported that the update of measuring equipment of CDC/CMA could result in uncertainty in the long-term trend analysis of *Rs* in China by introducing breaks. To eliminate this effect, the *Rs* data after 2000 were applied to build the model in our study. The daily ground-measured *Rs* data collected from 96 CDC/CMA stations during 2000‒2015 were used as target variables. The daily meteorological data used as predictors including air pressure, air temperature, wind speed, relative humidity, water vapor pressure, daily precipitation, and sunshine duration. The elevation data and cosine of the radian difference data at each station were also used as predictor variables. The daily *Rs* dataset was randomly split into two subsets for model construction: 80% for training the model, hence the rest 20% for assessing the performance of the ERT-based model. The k-fold cross-validation method was performed during the training procedure to evaluate the overall performance of the ERT-based model. The correlation coefficient (R), root-mean-square-error (RMSE), mean bias error (MBE), and mean relative error (MRE) were employed for evaluating the accuracy of the ERT-based model.

### 4.1. Evaluation Using Ground Measurements

**Figure 2:**
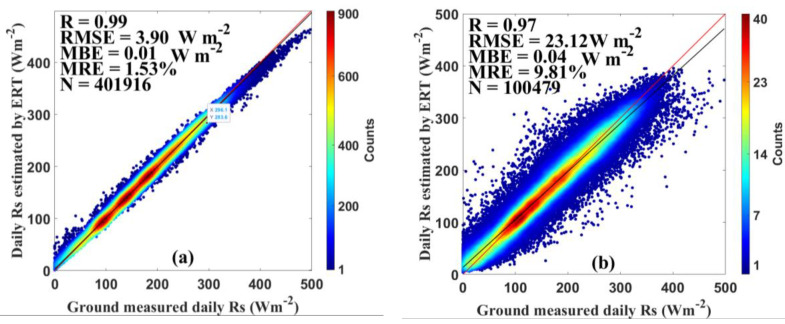
Evaluation results of the extremely randomized trees (ERT)-based model for the (**a**) training dataset and (**b**) test dataset against ground-measured *Rs* during 2000–2015.

The comparisons between the estimated daily *Rs* and corresponding ground measurements were conducted on national, regional, and station scales, respectively. The results of the ERT-based model for estimating *Rs* in the training and test stages are summarized in Table 1. The results on the national scale are displayed in Figure 2. For the training dataset, the ERT-based model obtained great performance with an overall R of 0.99, an RMSE of 3.90 Wm^−2^, an MBE of 0.01 Wm^−2^, and an MRE of 1.53%. Those values were 0.97, 23.12 Wm^−2^, 0.04 Wm^−2^, and 9.81% for the test dataset. It is clearly showed that both the training and test datasets correlated very well with ground measurements. On the regional scale, the range of the R, RMSE, MBE, and MRE values for the training and test datasets were 0.95–0.99, 3.38–24.63 Wm^−2^, −3.69–2.67 Wm^−2^, and 1.42–11.30%, respectively. On the station scale, the range of the R, RMSE, MBE, and MRE values for the training and test datasets were 0.89–0.99, 2.55–35.06 Wm^−2^, −9.19–9.00 Wm^−2^, and 0.86–16.95%, respectively. In the training stage, the ERT-based model had the RMSE, mean absolute errors (MAE), and MRE values lower than 30 Wm^−2^, 5 Wm^−2^, and 12% at all stations. While in the test stage, these statistics values were lower than 30 Wm^−2^, 5 Wm^−2^, and 12% at 76 out of 96 stations. The test results shown in the table indicate that the ERT-based model was accurate to obtain the *Rs* estimates.

**Figure 3:**
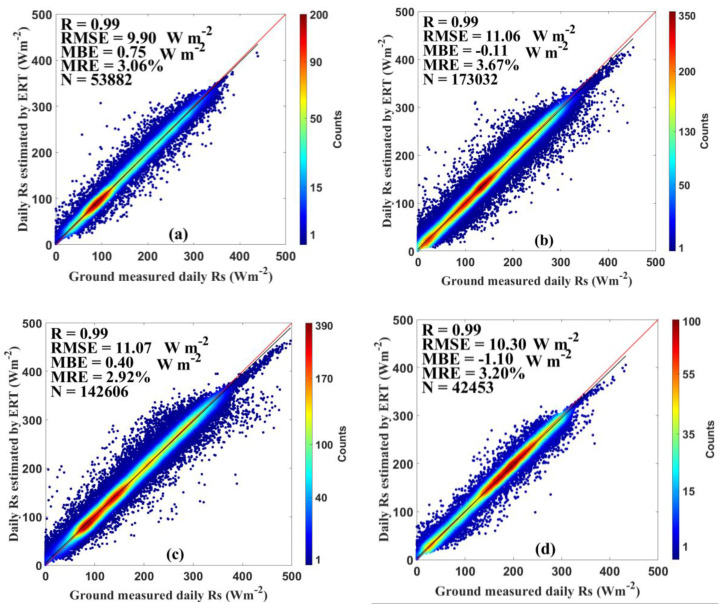

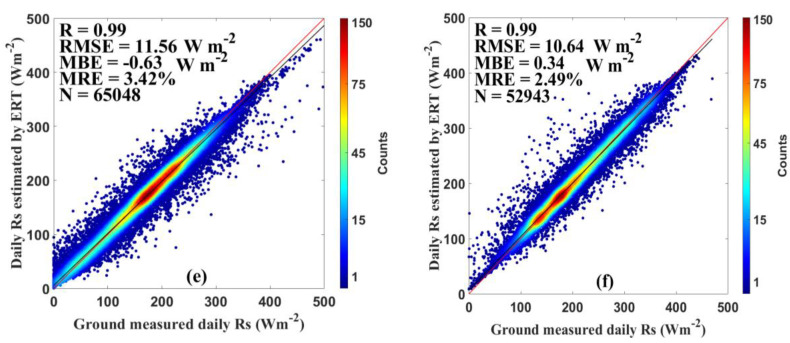
Evaluation results of the ERT-based model in (**a**) northeast China (NE), (**b**) east China (EC), (**c**) north China (NC), (**d**) south China (SC), (**e**) southwest China (SW), and (**f**) Tibetan Plateau (TP) during 2000‒2015.

**Figure 4:**
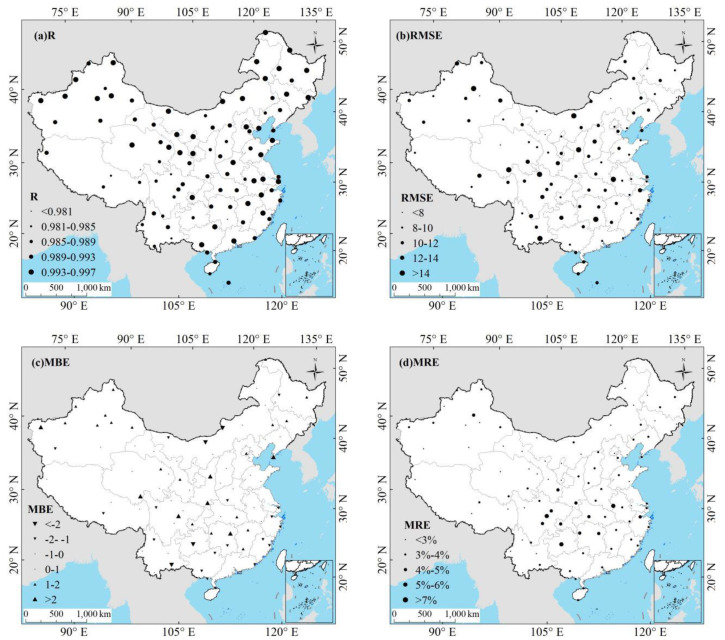
Spatial distribution of the ERT-based model performance on 96 CDC/CMA stations for (**a**) R, (**b**) RMSE, (**c**) mean bias error (MBE), and (**d**) mean relative error (MRE) during 2000–2015.

Figure 3 and Figure 4 show the estimation results using all datasets in both training and test stage to evaluate the overall performance of the ERT-based model. As shown in Figure 3, the test results suggest that the ERT-based model performed well in estimating the *Rs* in six climatic regions with reasonable accuracy. The ERT-based model in NE and TP provided slightly better accuracy than other regions, with R of 0.99 and 0.99, RMSE of 9.90 and 10.64 Wm^−2^, MBE of 0.75 and 0.34 Wm^−2^, and MRE of 3.06% and 2.49%, respectively. In addition to validations on national and regional scales, the validation of the ERT-based model was also conducted at individual CDC/CMA stations. As displayed in Figure 4, the daily estimated *Rs* agreed well with the *Rs* measurements at most CDC/CMA stations. For example, the great performance was found at station 52818 (Geermu) with R, RMSE, MBE, and MRE of 0.99, 7.23 Wm^−2^, −0.14 Wm^−2^ and 1.73%, respectively. In contrast, the ERT-based model did not provide great *Rs* estimates at station 57874 (Changning) with R, RMSE, MBE and MRE of 0.98, 19.90 Wm^−2^, −1.64 Wm^−2^, and 7.39%, respectively. Overall, the R values were 0.99 at 91 out of 96 stations. The RMSE, and MRE, values were lower than 15 Wm^−2^ and 5% at 92 stations. The MAE were lower than 2 Wm^−2^ at 86 stations. It is obvious that the ERT-based model successfully estimated accurate daily *Rs* at most CDC/CMA stations.

To assess the performance of the ERT-based model on different temporal scales, the validations on the daily and seasonal timescales were implemented as well. On the daily timescale, the range of the R, RMSE, MBE, and MRE values for the training and test datasets were 0.91–0.99, 2.23–32.97 Wm^−2^, −5.30–6.86 Wm^−2^, and 1.26–12.77%, respectively. The ERT-based model had the RMSE, MAE, and MRE values lower than 30 Wm^−2^, 5 Wm^−2^, and 12% on all days in the training stage. While in the test stage, these statistics values were lower than 30 Wm^−2^, 5 Wm^−2^, and 12% on more than 341 days. On the seasonal timescale, the range of the R, RMSE, MBE, and MRE values for the training and test datasets were 0.94–0.99, 2.75–28.47 Wm^−2^, −1.48–0.91 Wm^−2^, and 1.46–10.24%, respectively. The results in Table 1 indicate that the ERT-based model was promising to estimate the *Rs* accurately on both the daily and seasonal timescales.

**Figure 5:**
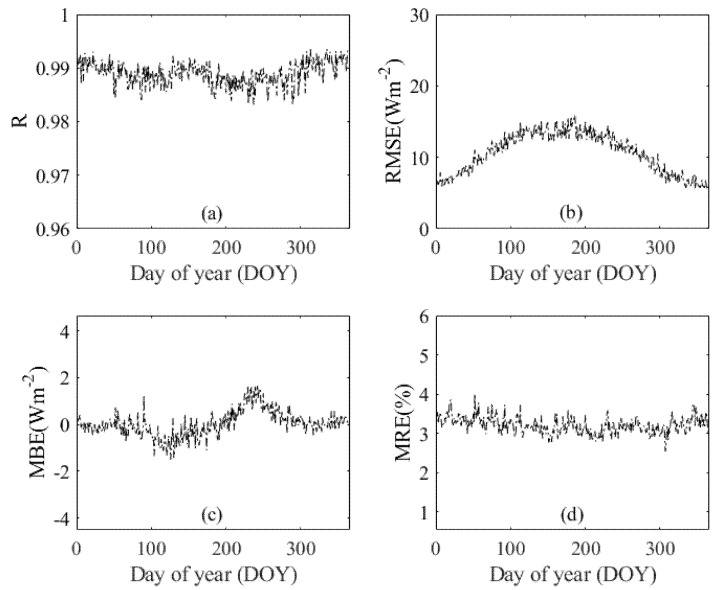
Daily performance of the ERT-based model (**a**) R, (**b**) RMSE, (**c**) MBE, and (**d**) MRE during 2000–2015.

**Figure 6:**
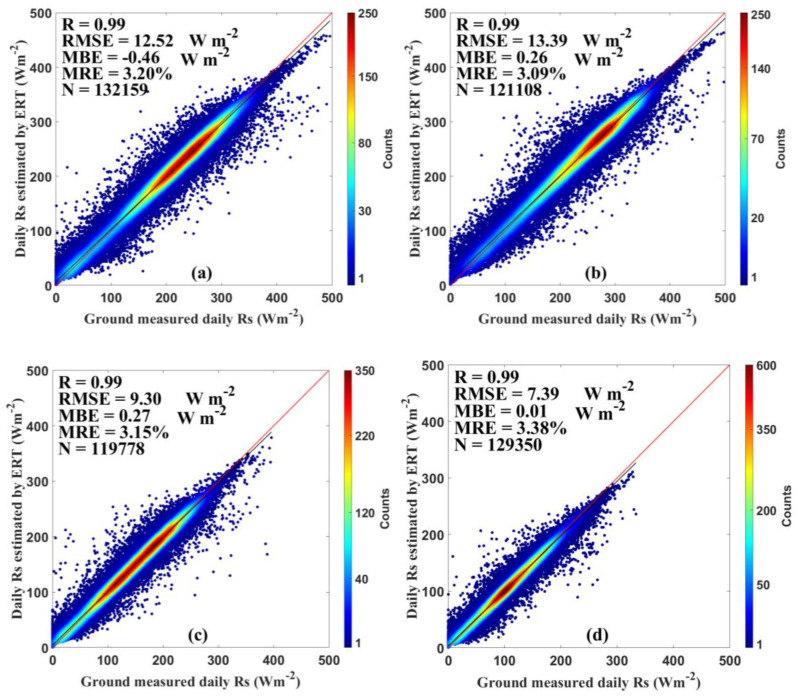
Evaluation results of the ERT-based model in (**a**) spring (March to May), (**b**) summer (June to August), (**c**) autumn (September to November), and (**d**) winter (December to February) over China during 2000–2015.

Figure 5 and Figure 6 demonstrate the estimation results using both the training and test datasets. As displayed in Figure 5, the performance of the ERT-based model was shown as a function of DOY at 96 CDC/CMA stations during 2000–2015. It illustrates that the ERT-based model performed well on most days. For example, the day 365 had relatively higher accuracy with R, RMSE, MBE, and MRE of 0.99, 5.53 Wm^−2^, 0.06 Wm^−2^, and 3.02%, respectively. In contrast, the ERT-based model did not give satisfactory *Rs* estimates on day 237 with R, RMSE, MBE, and MRE of 0.98, 13.74 Wm^−2^, 1.43 Wm^−2^, and 33.28%, respectively. Overall, the R values were higher than 0.98 on more than 345 days. The RMSE and MBE values were lower than 14 and 1 Wm^−2^ on more than 329 days. The MRE values were lower than 3.5% on more than 330 days. It demonstrates that the ERT-based model was capable of estimating the *Rs* with reasonable accuracy on most days in a year. Figure 6 shows the test results for the daily estimated *Rs* for different seasons during 2000–2015. The ERT-based model provided great accuracy in all seasons, especially in winter (DJF). The mean values of R, RMSE, MBE, MRE were 0.99, 7.39 Wm^−2^, 0.01 Wm^−2^, and 3.38% in winter, respectively.

**Figure 7:**
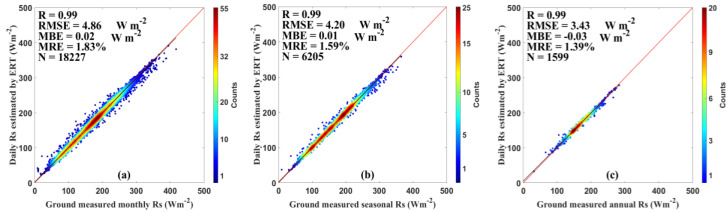
Evaluation results of the ERT-based model on the (**a**) monthly, (**b**) seasonal, and (**c**) annual timescales over China during 2000–2015.

The validation against ground measurements using all datasets in both training and test stages was also conducted on the synthetic timescales including the monthly, seasonal, and annual timescales (Figure 7). On the monthly timescale, the *Rs* estimates had an R of 0.99, an RMSE of 4.86 Wm^−2^, an MBE of 0.02 Wm^−2^, and an MRE of 1.83%. Those values were 0.99, 4.20 Wm^−2^, 0.01 Wm^−2^, and 1.59% at the seasonal timescale, and 0.99, 3.43 Wm^−2^, −0.03 Wm^−2^, and 1.39% at the annual timescale. These results suggest that synthetic datasets can capture spatiotemporal variations of *Rs* in China more accurately.

### 4.2. Spatiotemporal Analysis

#### 4.2.1. Spatial Variations

The above validation results show that the *Rs* estimates based on the ERT method are reasonably accurate compared to the ground measurements. Therefore, the ERT-based model was applied to reconstruct *Rs* data at 756 CDC/CMA stations during 1958−2015. The reconstructed *Rs* data at 454 CDC/CMA stations covering 46 consecutive years (1970–2015) were selected for analyzing trends of *Rs* in China.

**Figure 8:**
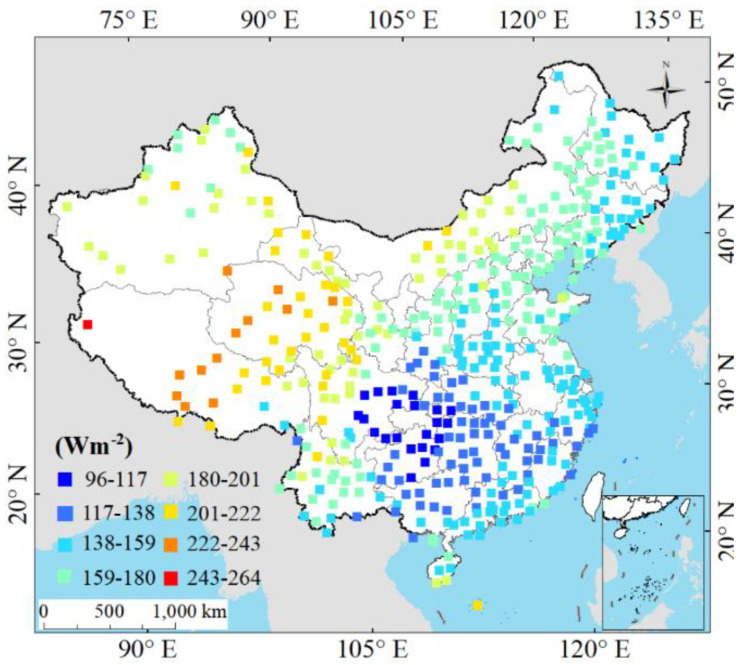
Spatial distribution of the annual mean *Rs* over China during 1970–2015.

The spatial distribution of annual mean *Rs* from 1970 to 2015 is displayed in Figure 8. It shows that *Rs* was higher in TP, NC, and NE than that in SW, EC, and SC. Apart from Sichuan and Guizhou, the *Rs* was spatially decreasing from western to eastern China, and the *Rs* in western China was decreasing with the increase of latitude. The TP had higher *Rs* values than other climatic regions. A previous study shows that the *Rs* in TP was the second highest globally and the highest in China [79]. Strong *Rs* in TP was mainly due to the small amount of cloud [29], rainfall, water vapor content [68], and good air transparency [80]. In contrast, the relatively lower *Rs* values were found in the Sichuan Basin, where the multiple hazes and low atmospheric transparency always happened due to the interactions between the cold and warm currents [81,82].

#### 4.2.2. Seasonal Variations

**Figure 9:**
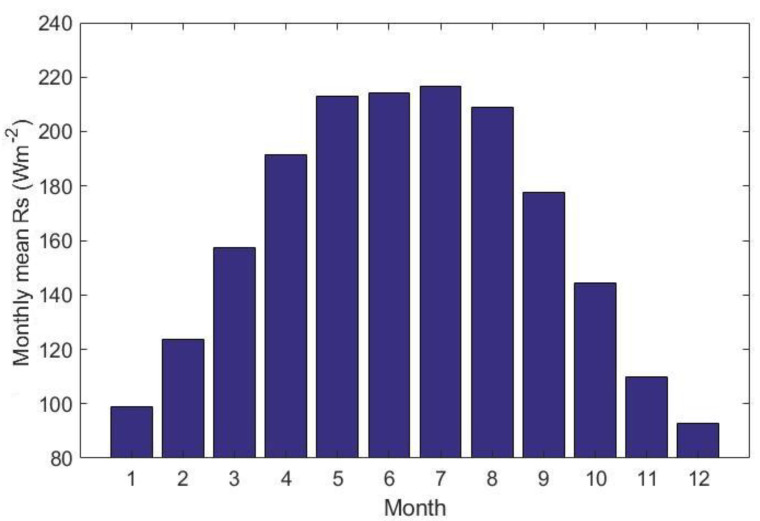
The monthly mean *Rs* over China during 1970–2015.

**Figure 10:**
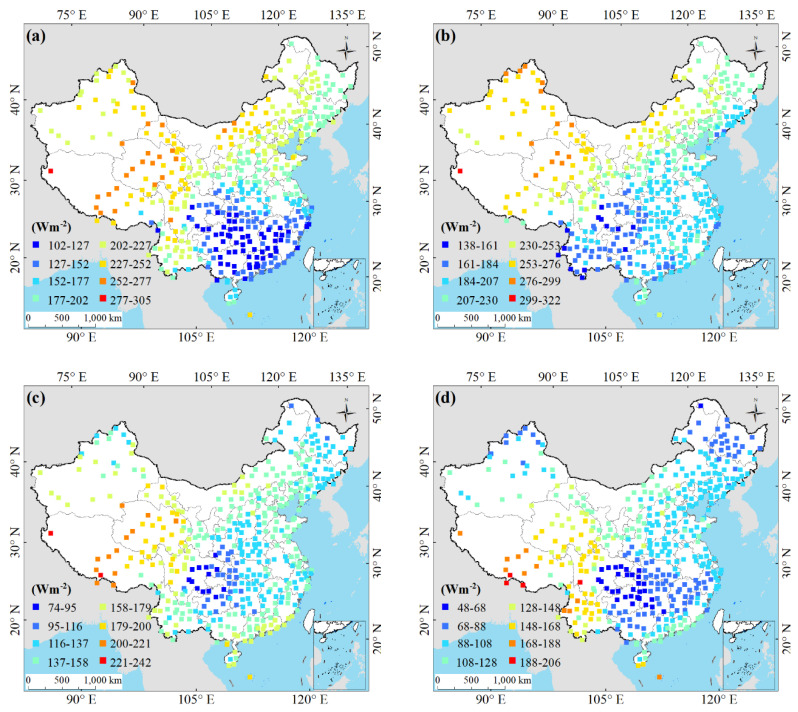
Spatial distribution of the seasonal mean *Rs* over China in (**a**) spring, (**b**) summer, (**c**) autumn, and (**d**) winter during 1970–2015.

The annual and seasonal mean *Rs* were obtained from individual stations with complete records observations. Figure 9 shows that the monthly *Rs* increased slowly from January to June then it declined gradually from July to December. This may relate to the changes of the annual cycle of solar zenith angle and the maximum sunshine duration over China. The monthly *Rs* in July was the largest with the value of 216.52 Wm^−2^, and the *Rs* in December was the smallest with the value of 92.73 Wm^−2^. The spatial distribution of the seasonal mean *Rs* over China between 1970 and 2015 is shown Figure 10. The average *Rs* in spring, summer, autumn, and winter were 187.22, 213.28, 143.99 and 105.06 Wm^−2^, respectively. The seasonal *Rs* trends detected by the M-K test in four seasons from 1970 to 2015 are shown in Table 2. The *Rs* showed an increasing trend in spring and decline trends in summer (*p* < 0.01), autumn (*p* < 0.01), and winter over China. In spring, the *Rs* in NE exhibited a significant decline (*p* < 0.01). Similarly, decline of *Rs* was also found in SW and TP. The seasonal *Rs* showed increasing trends in EC, NC, and SC. In summer, the significant decreasing trends were detected in *Rs* in EC (*p* < 0.01) and NE (*p* < 0.05). The decline tendencies of *Rs* were also found in NC and SC. The seasonal *Rs* showed increasing trends in SW and TP but were not significant. In autumn, the significant decline of *Rs* was found in EC (*p* < 0.01) and NC (*p* < 0.05). Similarly, the decline tendencies of *Rs* were also found in NE and SC but not significant. The increasing trends of *Rs* were detected in SW and TP. In winter, the *Rs* showed decline in all six climatic regions.

#### 4.2.3. Decadal Variations

**Figure 11:**
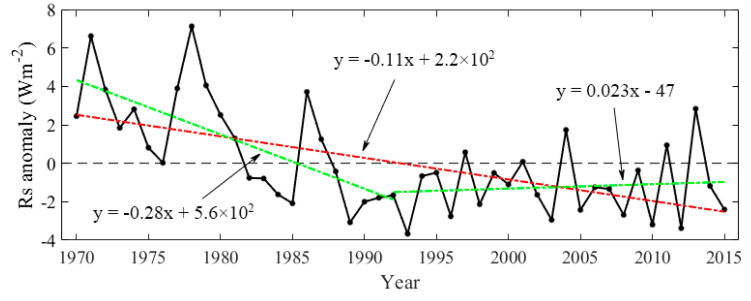
The anomaly of the annual mean *Rs* in China during 1970–2015. The green dashed lines are linear trends of 1970–1992 and 1992–2015.

Previous studies show that the *Rs* in China showed significant decline trends before the 1980s by 2 to 8 Wm^−2^ per decade [25,26,27,28,29,30,31,32], which did not persist into the 1990s. However, the magnitudes of variations in *Rs* were still controversial. The decadal variations of the *Rs* over China during 1970–2015 were analyzed based on the reconstructed *Rs* data at 454 CDC/CMA stations. As given in Figure 11, the *Rs* in China significantly decreased by −1.1 Wm^−^^2^ per decade (*p* < 0.01) during 1970–2015. The largest annual mean *Rs* value of 169.51 Wm^−^^2^ appeared in 1978, while the smallest value of 158.72 Wm^−^^2^ appeared in 1993. The trends of *Rs* during 1970–1992 and 1992‒2015 over China were also analyzed. The *Rs* significantly decreased (*p* < 0.01) from 1970 to 1992, by −2.8 Wm^−^^2^ per decade. While during 1992–2015, the *Rs* exhibited a recovery of 0.23 Wm^−^^2^ per decade.

**Figure 12:**
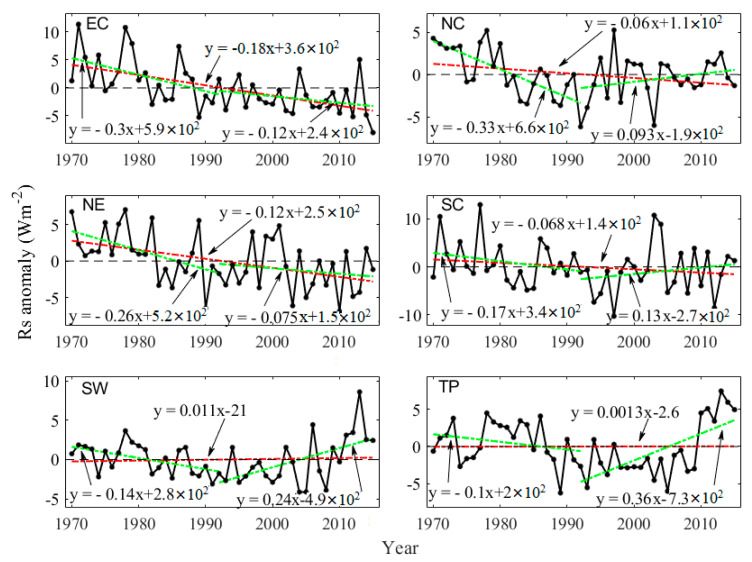
The anomaly of the annual mean *Rs* in six climatic regions during 1970–2015. The green dashed lines are linear trends of 1970–1992 and 1992–2015.

The decadal variations of *Rs* in six climatic regions during 1970–2015, 1970–1992, and 1992–2015 were also analyzed based on the reconstructed *Rs* dataset. As shown in Table 2 and Figure 12, the significant decreasing trends (*p* < 0.01) were detected in EC and NE during 1970–2015, decreased by −1.8 and −1.2 Wm^−2^ per decade. The *Rs* in NC and SC also showed the decreasing trends (−0.6 and −0.7 Wm^−2^ per decade, respectively) during 1970–2015. Moreover, the increasing trends were detected in SW (0.1 Wm^−2^ per decade) and TP (0.01 Wm^−2^ per decade) during 1970–2015. Between 1970 and 1992, the *Rs* showed significant decline trends (*p* < 0.05) in NC, NE, and SW (−3.3, −2.6, and −1.4 Wm^−2^ per decade, respectively). The decline trends of *Rs* were also detected in EC, SC, and TP (−3.0, −1.7, and −1.0 Wm^−2^ per decade, respectively) but not significant. From 1992 to 2015, the *Rs* in SW and TP showed significant increasing trends (2.4 and 3.6 Wm^−2^ per decade, respectively). Similarly, increasing trends were also found in NC and SC (0.93 and 1.3 Wm^−2^ per decade) but were not significant. However, the *Rs* showed decreasing tendencies of −1.2 and −0.75 Wm^−2^ per decade in EC and NE, respectively.

**Figure 13:**
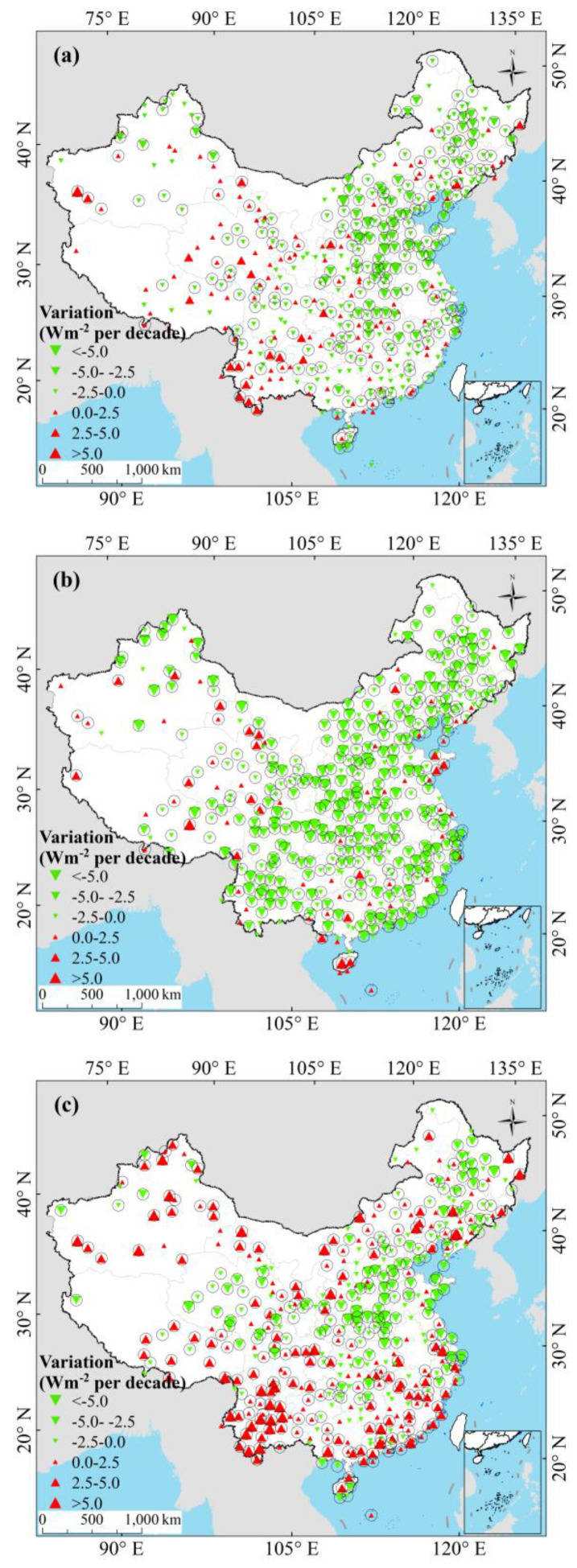
Decadal variation of the annual mean *Rs* on 454 stations during (**a**) 1970–2015, (**b**) 1970–1992, and (**c**) 1992–2015. Trends at the 95% significance level (*p* < 0.05) are denoted by five-pointed black circle symbols.

The decadal variations of *Rs* at 454 CDC/CMA stations were shown in Figure 13. Among 454 CDC/CMA stations used in this study, there were 63, 147, 95, 42, 66, and 41 stations in NE, EC, NC, SC, SW, and TP, respectively. The *Rs* exhibited the decreasing trends at 317 (70%) stations during 1970‒2015 (Figure 13a). During 1970‒2015, the *Rs* showed decline at 52 (83%), 125 (85%), 61 (64%), 30 (71%), 32 (48%), and 17 (41%) stations in NE, EC, NC, SC, SW, and TP, respectively. The *Rs* exhibited decline at more than 64% of the stations in NE, EC, NC, SC. On the contrary, the *Rs* showed the increasing trends at more than 50% of the stations in SW and TP. From 1970 to 1992 (Figure 13b), the *Rs* showed the decreasing trends at 378 (83%) stations. The *Rs* showed the decline trends at 58 (92%), 128 (87%), 78 (82%), 27 (64%), 59 (89%), and 28 (68%) stations in NE, EC, NC, SC, SW, and TP, respectively. The *Rs* exhibited the decline trends at more than 64% of the stations in six climatic regions. Between 1992 and 2015 (Figure 13c), the *Rs* showed the increasing trends at 233 (51%) stations over China, including 20 (32%), 55 (37%), 60 (63%), 27 (64%), 51 (77%), and 20 (49%) stations in NE, EC, NC, SC, SW, and TP, respectively. It shows that the increasing trends of *Rs* mainly concentrated in NC, SC, SW, and TP during 1992–2015. All in all, the *Rs* showed the decline trends at more than 70% of the CDC/CMA stations during 1970–1992 and 1970–2015, while it showed the increasing trends at more than 51% of the stations during 1992–2015.

## 5. Discussion

To investigate the influence of input variables of the ERT-based model on the estimation results, the variable importance measures provided by the ERT method was conducted. As shown in Table 3, the importance of the input variables of the ERT-based model was in the order of sunshine duration, cosine of the radian difference, air pressure, water vapor pressure, relative humidity, elevation, air temperature, wind speed and daily precipitation. The sunshine duration had significant influence on estimating *Rs*, while the wind speed and daily precipitation had relative low influence on the *Rs* estimation.

**Figure 14:**
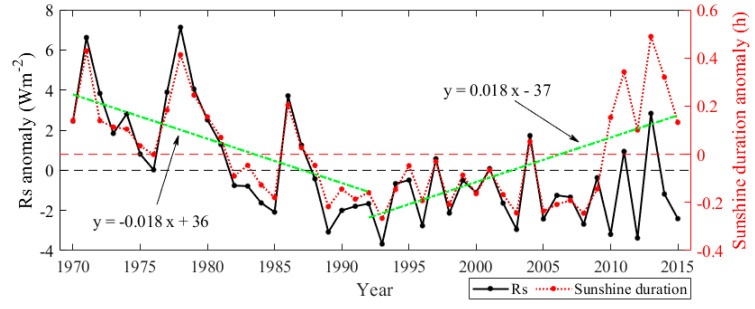
Comparison of the anomaly of the annual mean *Rs* and sunshine duration between 1970 and 2015. The green dashed lines are linear trends of 1970–1992 and 1992–2015.

**Figure 15:**
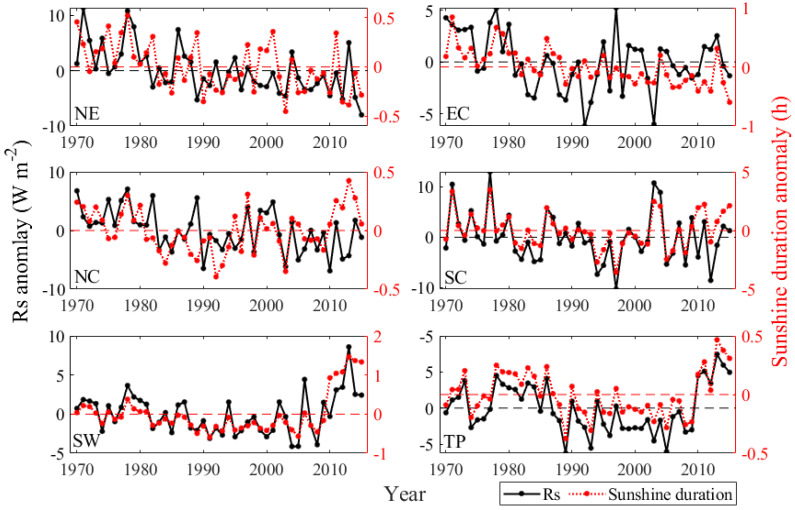
Comparison of the anomaly of the annual mean *Rs* and sunshine duration between 1970 and 2015 in six climatic regions.

The above analysis reveals significant declining tendencies of the *Rs* in China during 1970–2015. Meanwhile, there was a significant decline during 1970–1992, and a recovery during 1992–2015. Previous studies pointed that the sunshine duration decreased by −0.17 h per decade in China during 1960–2000 [83,84], the variations of the *Rs* in China may relate to the variations of sunshine duration. The variable importance measures indicate that the sunshine duration had significant influence on the *Rs* estimation. To further investigate probable causes of changes in *Rs*, the comparison of the variations of sunshine duration and *Rs* was conducted. Figure 14 shows the anomalies of the annual mean *Rs* and sunshine duration in China during 1970–2015, 1970–1992, and 1992–2015, respectively. It is clear that annual variations of the anomaly of *Rs* were almost in line with variations of sunshine duration from 1970 to 2015, before 1992 the sunshine duration exhibited a decreasing trend (0.18 h per decade), and an increasing trend (0.18 h per decade) afterward. As shown in Figure 15, it illustrates that the trends of sunshine duration were consistent with the trends of the *Rs* in six climatic regions from 1970 to 2015. The consistency of variations in sunshine duration and *Rs* indicates that the variations of *Rs* were likely due to long-term variations of sunshine duration. In the most recent years, a slight difference between the sunshine duration and the *Rs* was observed. This probably contributes to the influence of the aerosol variations on *Rs* according to the previous studies [37,48,85,86,87,88]. For instance, Jia et al. [87] reported that the variations in *Rs* is likely due to the variations in the aerosols, cloud, and water vapor in north and south China. Wang and Pinker [85] found that the global average aerosol showed an increasing trend during 1979–2006, particularly in east and south Asia (including China). The increase in global average aerosol could be associated with a continuous decrease of *Rs* in China from 1979 to 2006. Qin et al. [86] pointed out that *Rs* over China declined gradually between 1980 and 2015, which may relate to the increasing aerosol radiative forcing effects over China in recent decades. It is clear that the spatiotemporal variations in *Rs* over China were complicated due to the effects of natural factors and human activities. The influence of other factors (e.g., aerosols, cloud, and topography) on *Rs* estimation are still needed to be investigated in future research.

## 6. Conclusions

In this study, an *Rs* estimation model was constructed employing the ERT algorithm based on the quality-controlled daily *Rs* data and routine meteorological data. The accuracy and applicability of the ERT-based model in estimating daily *Rs* over China were investigated. A new daily *Rs* dataset was developed at 756 CDC/CMA stations from 1958 to 2015. Moreover, the spatial and temporal variations of the *Rs* at 454 CDC/CMA stations covering 46 consecutive years (1970–2015) were analyzed. The results indicate that the ERT-based model estimated well the *Rs* at the national scale with R of 0.97, RMSE of 23.12 Wm^−2^, MBE of 0.04 Wm^−2^, and MRE of 9.81%. The ERT-based model also performed well in estimating the *Rs* at most CDC/CMA stations and individual days. Furthermore, the spatiotemporal variations in the *Rs* over China were investigated using the M-K test based on the reconstructed dataset. During 1970–2015, the annual mean *Rs* in China showed a significant decline (*p* < 0.01) by −1. 1 Wm^−2^ per decade. The *Rs* decreased significantly (*p* < 0.01) in EC and NE by −1.8 and −1.2 Wm^−2^ per decade. The *Rs* in NC and SC also showed decreasing trends while the *Rs* in SW and TP showed increasing trends. The *Rs* showed decreasing trends at 317 out of 454 CDC/CMA stations. During 1970–1992, the *Rs* showed a significant decreasing trend (−2.8 Wm^−2^ per decade) in China. The declined trends of *Rs* were detected at 378 out of 454 CDC/CMA stations. During 1992–2015, the *Rs* in China showed a recovery (0.23 Wm^−2^ per decade). The increasing trends of *Rs* were detected at 233 out of 454 CDC/CMA stations, which are mainly concentrated in NC, SC, SW, and TP. The comparison of *Rs* and sunshine duration illustrates that the variations of the *Rs* in China may relate to the variations in sunshine duration. More ground measurements of *Rs* and other important factors including aerosols, clouds, water vapor should be collected for further studies to investigate the variations of *Rs* over China.

Overall, the reconstruction of new daily long-term *Rs* with high accuracy would be a useful data source for the related climate change studies. More attention needs to be paid to quantitative analysis of *Rs* and other climatic factors.

## Figures and Tables

**Table 1:** Training and test results of the ERT-based model.

Scale.	Dataset		R	RMSE	MBE	MRE
National	Train		0.99	3.9	0.01	1.53%
	Test		0.97	23.12	0.04	9.81%
Regional	Train	NE	0.99	3.38	0.28	1.42%
		EC	0.99	3.39	−0.05	1.74%
		NC	0.99	4.02	0.2	1.42%
		SC	0.99	3.63	−0.51	1.53%
		SW	0.99	4.05	−0.28	1.64%
		TP	0.99	3.86	0.12	1.23%
	Test	NE	0.97	21.13	2.67	9.61%
		EC	0.96	23.35	−0.33	11.30%
		NC	0.97	23.41	1.2	8.91%
		SC	0.96	21.97	−3.49	9.95%
		SW	0.95	24.63	−2.04	10.64%
		TP	0.95	22.52	1.18	7.60%
Seasonal	Train	Spring	0.99	4.5	−0.21	1.53%
		Summer	0.99	4.66	0.1	1.46%
		Autumn	0.99	3.36	0.14	1.52%
		Winter	0.99	2.75	−0.01	1.66%
	Test	Spring	0.95	26.58	−1.48	9.89%
		Summer	0.94	28.47	0.91	9.66%
		Autumn	0.96	19.65	0.8	9.58%
		Winter	0.96	15.57	0.07	10.24%
Stational	Train		0.99	2.55–7.54	−1.42–1.22	0.86–3.66%
	Test		0.89–0.98	14.95–35.06	−9.19–9.00	4.95–16.95%
DOY	Train		0.99	2.23–5.38	−0.73–0.76	1.26–1.86%
	Test		0.91–0.97	11.32–32.97	−5.30–6.86	7.55–12.77%

The unit of RMSE and MBE is Wm^−2^.

**Table 2:** Values of Z for the Mann–Kendall (M-K) test.

Region	Annual (1970–2015)	Annual (1970–1992)	Annual (1992–2015)	Spring	Summer	Autumn	Winter
China	−3.79 **	−3.43 **	0.12	0.09	−4.60 **	−2.63 **	−1.76
EC	−4.11 **	-1.90	−1.81	0.68	−4.56 **	−2.63 **	−1.65
NC	−1.69	−3.27 **	0.77	0.32	−1.72	−2.25 *	−1.93
NE	−3.03 **	−2.59 **	−0.62	−2.58 **	−2.42 *	−1.12	−1.86
SC	−1.16	−1.00	0.87	0.83	−1.31	−0.83	−0.66
SW	1.38	−2.32 *	2.46 *	−0.38	1.40	1.04	−0.49
TP	1.26	−1.11	2.51 *	−0.86	0.73	1.88	−0.57

* Trend at the 95% significance level (*p* < 0.05); ** Trend at the 99% significance level (*p* < 0.01).

**Table 3:** Variable importance measure.

Variable	Importance	Variable	Importance
Sunshine duration	0.47	Elevation	0.04
Cosine of the radian difference	0.16	Air temperature	0.04
Air pressure	0.13	Wind speed	0.01
Water vapor pressure	0.07	Daily precipitation	0.01
Relative humidity	0.07		
